# The Thames

**Published:** 1858-07

**Authors:** 


### THE THAMES.

There has been no small excitement in London during the late hot weather, in regard to the state of the Thames. The Houses of Parliament have become infested with strange smells. Justice, in its court, has hesitated whether to proceed, lest the Oxford Black Assize be reperpetrated; and royalty on the river has found a bouquet something more than a luxury. There have been strange stories flying of men struck down with the stench, and of all kinds of fatal diseases upspringing on the river's banks. There has been much exaggeration as to the amount of mischief done to the health of the community by the foul river, and much absurd speculation as to the necessary consequences to be expected from it. Plague is predicted, yellow fever, and cholera. The two latter might come if they were brought; and if they did come by their poisons being conveyed, their spread would be unquestionably favoured by the present impure state of things. These diseases, however, need not be expected from a river, however dirty, if it be not charged with their poisons. The true risk of an epidemic from the river in its present condition lies mainly in a risk of fever in persons living on or near the river, and who, heedless of all law of cleanliness, take their water supply from this filthy source. At the same time, there can be no doubt that a contagious epidemic of any kind breaking out now in the neighbourhood of the Thames would prove irresistibly fatal, since the gaseous emanations from a great sewer eat up the disinfecting ozone, and inhaled by animal bodies derange the organic chemistry, and favour the development of disease. Without any unnecessary exaggeration of the evil, it is necessary therefore that the Thames should be cleansed, and that such arrangements should be made as shall at once lead to the constant and ready removal of the sewage with which the water is loaded. Of the thousand schemes for Thames purification, all are alike inapplicable in an emergency, and, as we still believe, inapplicable altogether; the plan suggested by the late Royal Commission being no exception to this rule. Amongst the temporary plans for relieving the river of its dirty burthen, the one most likely to answer the purpose for the present, and it may be for the future, consists in adopting for the direct removal of sewage a system resembling the present water conveyance system. A series of pipes laid down in the riverside to receive the sewage flow, and convey it onwards towards the sea, from the city, would at once meet the emergency. A series of small sewer streams would thus be laid down in the midst of the purer river current, but separated from it; and this system of iron sewerage might be afterwards extended, removed, or modified, as required. The iron canals could be flushed from the river itself, by an extension of them upwards to a distance beyond the first main source of contamination, and by conveying the contents of the sewers into them by side intercepting branches. The plan here proposed is not new. It is embraced in a scheme laid down by Mr. Austin. It is described on a grand scale in our last number, in the plan suggested by Dr. Huxley. The modification, indeed, in the present suggestion, consists only in substituting a series of small canals for one or two of massive proportions; but its advantages are obvious; it admits of being carried out at a comparatively trifling cost, with great rapidity, and as a grand experiment which may be given up, should it fail, and no one feel the worse for the adventure.

There is yet another idea which has occurred to us, and which, at least, deserves as much consideration as the majority of the schemes which have been brought before the public. This idea suggests that floating reservoirs might be constructed for the reception of the sewer flow. We see no reason why the contents of a sewer might not be intercepted by a floating reservoir, through which the water part of the sewage might filter, and which, when charged with the solid and valuable sewage matter, might be tugged away for its contents to be disembarked elsewhere, and disposed of for agricultural purposes. We doubt not that with this arrangement rendered practicable, all the cost of sewage removal would be undertaken by, and could be safely entrusted to private enterprize. The lading of sewage vessels, with valuable cargoes indeed, introduces merely a new business for the river sailor.

